# 

**Published:** 1950-01

**Authors:** 


### The Bristol
### Medico-Chirurgical Journal

A JOURNAL OF THE MEDICAL SCIENCES FOR THE

### West of England and South Wales

PUBLISHED UNDER THE AUSPICES OF

*THE BRISTOL MEDICO-CHIRURGICAL SOCIETY*

EDITOR

E. WATSON-WILLIAMS, *M.C.*, M.D., Ch.M., F.R.C.S.

WITH WHOM ARE ASSOCIATED

G. E. F. SUTTON, *M.C.*, M.D., F.R.C.P.

J. H. MIDDLEMISS, M.D., D.M.R.D., F.F.R.

N. S. CRAIG, *M.C.*, M.D., *Assistant Editor*

"Scire est nescire, nisi id me

Scire alius scire."

1950 *VOL. LXVII*

BRISTOL: J. W. ARROWSMITH LTD.

### 1950 Contents of Vol. LXVII

*Page*

Medicine in Eighteenth-Century Bristol. By H. J. Orr-Ewing, F.R.C.P. 1

The Thirty-Eighth Long Fox Memorial Lecture: The Use of Tracer Elements in Biology and Medicine. By John E. Harris .. .. 10

Some Post-Mortem Surprises. By F. D. M. Hocking, M.Sc., M.B., F.R.I.C. 17

Some Medical Problems in China To-day. By Coralie Rendle-Short, M.B., Ch.M., M.R.C.O.G. .. .. .. .. .. .. 22

Poliomyelitis in Bristol, 1949. By James Macrae, M.D., F.R.F.P.S., D.P.H. 43

Epidemic Myalgia—Bornholm Disease. By N. S. Craig, *M.C.*, M.D. .. 50

Gastric Ulcer with Perforation into the Lesser Sac: *Report of a Case.* By H. A. Vere Nicoll, F.R.C.S. .. .. .. .. .. .. 53

Congenital Atresia of the Oesophagus : *Report of three cases.* By Ronald Belsey, F.R.C.S. .. .. .. .. .. .. .. .. 54

Some Recent Advances in Paediatrics. By Wilfrid P. H. Sheldon, M.D., F.R.C.P. .. .. .. .. .. .. .. .. .. 56

The Work of a Department of Industrial Health. By R. C. Browne, D.M., M.R.C.P. .. .. .. .. .. .. .. .. .. 67

The General Use of Histamine Antagonists. By Robert P. Warin, M.D., M.R.C.P. .. .. .. .. .. .. .. .. .. 75

Impressions of American Rheumatology and Recent Advances in " Rheumocrinology ". By G. D. Kersley, *T.D.*, M.D., M.A., F.R.C.P. .. 82

The Value of X-Ray Therapy for Metastases in Bone, from Carcinoma of the Breast. By Sydney Curwen, D.M.R.T. .. .. .. .. 88

The Prognosis in Hypertension. By D. H. Davies, M.D., M.R.C.P. .. 103

The Surgical Treatment of Hypertension. I—By G. L. Alexander, F.R.C.S.; II—By Ashton Miller, F.R.C.S. .. .. .. .. 110

Editorial Notes .. .. .. .. .. .. .. 28, 92, 120

Reviews of Books .. .. .. .. .. .. 30, 59, 94, 120

Obituary .. .. .. .. .. .. .. .. .. .. 29, 93

Meetings of Societies .. .. .. .. .. .. 34, 61, 96, 124

Local Medical Notes .. .. .. .. .. .. 39, 65, 100, 126

### 1950 Index to Vol. LXVII

Alexander, G. L.—The Surgical Treatment of Hypertension, 110

Atresia of the Oesophagus, Congenital. Ronald Belsey, 54

Belsey, Ronald.—Congenital Atresia of the Oesophagus: *Report of three cases*, 54

Bornholm Disease—Epidemic Myalgia. N. S. Craig, 50

Bristol, Medicine in Eighteenth Century.—H. J. Orr-Ewing, 1

Bristol, 1949, Poliomyelitis in.—James Macrae, 43

Browne, R. C.—Work of a Department of Industrial Health, 67

Carcinoma of the Breast, The Value of X-Ray Therapy for Metastases in Bone from.—Sydney Curwen, 88

China To-day, Some Medical Problems in.—Coralie Rendle Short, 22

Congenital Atresia of the Oesophagus: *Report of three cases*.—Ronald Belsey, 54

Craig, N. S.—Epidemic Myalgia—Bornholm Disease, 50

Curwen, Sydney.—The Value of X-Ray Therapy for Metastases in Bone from Carcinoma of the Breast, 88

Davies, D. H.—Prognosis in Hypertension, 103

Devaluation—Editorial Note, 28

Editorial Notes, 28, 92, 120

Epidemic Myalgia—Bornholm Disease. —N. S. Craig, 50

Gastric Ulcer with Perforation into the Lesser Sac: *Report of a Case*.—J. A. Vere Nicoll, 53

General Use of Histamine Antagonists. —Robert P. Warin, 75

Harris, John E.—The Thirty-Eighth Long Fox Memorial Lecture: The Use of Tracer Elements in Biology and Medicine, 10

Histamine Antagonists, General Use of. —Robert P. Warin, 75

Hocking, F. D. M.—Some Post-Mortem Surprises, 17

Hypertension — Prognosis: D. H. Davies. Surgical Treatment: I, G. L. Alexander; II, Ashton Miller, 110

Impressions of American Rheumatology and Recent Advances in " Rheumocrinology ".—G. D. Kersley, 82

Industrial Health, Work of a Department of.—R. C. Browne, 67

Kersley, G. D.—Impressions of American Rheumatology and Recent Advances in " Rheumocrinology ", 82

Local Medical Notes, 39, 65, 100, 126

Long Fox Memorial Lecture, the Thirty-Eighth, 10

Macrae, James.—Poliomyelitis in Bristol, 1949, 43

Medicine in Eighteenth - Century Bristol.—H. J. Orr-Ewing, 1

Metastases in Bone from Carcinoma of the Breast, Value of X-Ray Therapy for.—Sydney Curwen, 88

Miller, Ashton.—The Surgical Treatment of Hypertension, 115

Obituary: Frank Gower Bergin, 29; Ronald Grey Gordon, 92

Oesophagus, Congenital Atresia of the. R. Belsey, 54

Orr - Ewing, H. J. — Medicine in Eighteenth-Century Bristol, 1

Paediatrics, Some Recent Advances in. Wilfrid P. H. Sheldon, 56

Poliomyelitis in Bristol, 1949.—James Macrae, 43

Post-Mortem Surprises, Some.—F. D. M. Hocking, 17

Prognosis in Hypertension.—D. H. Davies, 103

Rendle-Short, Coralie.—Some Medical Problems in China To-day, 22

Reviews of Books, 30, 59, 94, 120 ; Editorial, 120

Rheumatology, Impressions of American, and Recent Advances in Rheumocrinology.—G. D. Kersley, 82

Sheldon, Wilfrid P. H.—Some Recent Advances in Paediatrics, 56

Societies, Meetings of:
- Bristol M.-Chir. Soc., 34, 62, 96, 124
- B.M.A.:
  - Bath, Bristol and Somerset Branch, 38, 64, 99
  - Gloucestershire Branch, 38, 126
  - Bristol Division, 39, 64, 99, 126
  - Newport Division, 99
  - West Somerset Division, 39
- British Medical Guild, 61
- British Orthopaedic Assn., 35
- Cardiff Medical Society, 124
- Clinical Soc. of Bath, 97, 124
- Cornwall Clinical Soc., 98, 126
- Devon and Exeter Med.-Chir. Soc., 36, 61, 125
- Plymouth Med. Soc., 98
- S.W. Laryngological Assn., 37, 98
- S.W. Radiological Soc., 98
- Torquay and District Med. Soc., 64, 125.
- Wessex Rahere Club, 38, 99, 126
- West of England and Wales Soc. Dermatol., 37
- West Somerset Med. Club., 98

Some Medical Problems in China To-day.—Coralie Rendle-Short, 22

Some Post-Mortem Surprises.—F. D. M. Hocking, 17

Some Recent Advances in Paediatrics.—Wilfrid P. H. Sheldon, 56

Surgical Treatment of Hypertension, The.—I, G. L. Alexander, II, ; Ashton Miller, 110.

Tracer Elements in Biology and Medicine, Use of.—John E. Harris, 10

To Account Rendered.—Editorial Note, 92

Use of Tracer Elements in Biology and Medicine, The: The Thirty-Eighth Long Fox Memorial Lecture.—John E. Harris, 10

Vere Nicoll, J. A.—Gastric Ulcer with Perforation into the Lesser Sac, 53

Warin, Robert P.—General Use of Histamine Antagonists, 75

Work of a Department of Industrial Health.—R. C. Browne, 67

X-Ray Therapy for Metastases in Bone from Carcinoma of the Breast.—Sydney Curwen, 88

### Local Medical Notes

### Appointments

**South-Western Regional Hospital Board.**—

A. G. McPherson, M.Chir., F.R.C.S.—Consultant Surgeon, Bristol.

J. M. Naish, M.D., M.R.C.P.—Consultant Physician, Bristol.

D. A. Barley, M.B., F.R.C.S., D.L.O.—Consultant Ear, Nose, and Throat Surgeon, Plymouth.

J. J. Kempton, M.B.E., M.D., M.R.C.P., D.C.H.—Paediatrician, Canadian Red Cross Memorial Hospital.

**University of Birmingham.**—

S. D. Loxton, Lecturer in Obstetrics and Gynaecology.

### SIR ALEXANDER FLEMING*

In honouring Sir Alexander Fleming we do more than acclaim a great scientist; we convey our thanks to one whose work has been of the utmost benefit to mankind. There will be many present in this hall who have reason to be profoundly grateful to him for health and even for life itself.

Sir Alexander Fleming was associated in his early work at St. Mary's Hospital with Sir Almroth Wright, a pioneer in the method of combating disease by stimulating the defences of the body. Sir Alexander was the first to realize that an " antiseptic " must be not only toxic to bacteria, but harmless to their natural enemies the white blood corpuscles.

After pioneering work on antiseptics during and after the First World War, when he served as a Captain in the R.A.M.C., Sir Alexander made the remarkable discovery that human tears contain a substance, lysozyme, capable of destroying bacteria. Lysozyme was later found in white of egg, and although its nature has only recently been determined this work drew attention to natural antibacterial agents.

Such in brief, was the background when an unexpected event occurred twenty-one years ago in the bacteriological laboratory of St. Mary's Hospital, Paddington. A drifting spore of a mould, *penicillium notatum*, came to rest upon a culture plate of bacteria. That spore grew to a patch of mould, and Sir Alexander noticed that the neighbouring bacteria had not developed as they had over the rest of the plate. He later remarked with characteristic caution " the appearance of that culture plate was such that I thought it should not be neglected ".

It was not neglected; pure cultures of the mould were grown; the active substance produced was named " penicillin ", and its value as a promising antiseptic was recognized. The true worth of penicillin could not, however, be seen at that time because it could not be isolated. It was not until the early 1940s that Sir Alexander's work was brought to fruition at Oxford in the hands of Sir Howard Florey, Dr. Chain, Dr. Abraham and others.

This is not the occasion to recount the full story of the use of penicillin in the treatment of war wounds and in the case of otherwise fatal bacterial infections. Nor is it the time to tell of the unprecedented collaborative investigation between some forty research laboratories in this country and in the United States of America. This collaboration led both to the production of the large quantities of penicillin necessary for the treatment of war casualties, and to the establishment of the chemical structure of this remarkable and elusive substance. Seldom can one man have started scientific investigations of such importance and of such magnitude.

Sir Alexander has, nevertheless, reasons for disappointment. Chemists have not been able to improve penicillin. Nor have chemists been able to produce it without the mould, save in minute traces. Again, bacteria can unfortunately become resistant to penicillin; it is therefore necessary that the sale of this beneficent material should be restricted. This is one of those rare controls which we willingly accept; it is a control which even you, Mr. Chancellor, may be disposed to retain.

In his work Sir Alexander Fleming may be said to have begun the

* Professor Baker's speech commending Sir Alexander Fleming for the degree of Doctor of Science, *Honoris Causa*, in the University of Bristol, 19th October, 1949.

writing of a great new book. The first chapters bear the titles " Lysozyme" and " Penicillin ". Other workers are writing later sections, " Gramicidin ", " Streptomycin ", " Chloromycetin ", " Aureomycin ". But whenever the final chapter is written, the inspiration for the whole volume will still be found in the opening pages.

### Degrees and Diplomas

**University of Bristol.**—

M.B., Ch.B.—Diana F. J. Duncan, A. R. Ebanks, Dorothy Field, Margaret J. Francis, G. C. K. Herapath, Ellen M. Marron, Jean Musgrave, Margaret R. Salisbury.

L.D.S.—D. C. Berry, G. D. Pamely.

D.P.M.—Muriel J. Davies.

**University of Cambridge.**—
M.D.—H. R. E. Wallis.

**University of London.**—
M.D.—D. G. Stewart.

**Royal College of Physicians**—
M.R.C.P.—F. Bolton.

**Royal College of Surgeons.**—
D.M.R.D.—W. R. Cole.

**Conjoint Board.**—
M.R.C.S., L.R.C.P.—Mrs. Dorothy Field.

